# Excitation-Power-Dependent Upconversion Luminescence Competition in Single β-NaYbF_4_:Er Microcrystal Pumped at 808 nm

**DOI:** 10.1186/s11671-021-03649-1

**Published:** 2022-03-26

**Authors:** Maohui Yuan, Zining Yang, Xu Yang, Linxuan Wang, Rui Wang, Sheng Lan, Kai Han, Hongyan Wang, Xiaojun Xu

**Affiliations:** 1 College of Advanced Interdisciplinary Studies, National University of Defense Technology, Changsha, 410073 China; 2 State Key Laboratory of Pulsed Power Laser Technology, National University of Defense Technology, Changsha, 410073 China; 3 Hunan Provincial Key Laboratory of High Energy Laser Technology, National University of Defense Technology, Changsha, 410073 China; 4 Department of Physics and Chemistry, PLA Army Academy of Special Operations, Guangzhou, 510507 China; 5 Guangdong Provincial Key Laboratory of Nanophotonic Functional Materials and Devices, School of Information and Optoelectronic Science and Engineering, South China Normal University, Guangzhou, 510006 China

**Keywords:** Single NaYbF_4_:Er microcrystals, Upconversion luminescence, 808 nm excitation, Excitation-power-dependent, Multicolor

## Abstract

Controlling the upconversion luminescence (UCL) intensity ratio, especially pumped at 808 nm, is of fundamental importance in biological applications due to the water molecules exhibiting low absorption at this excitation wavelength. In this work, a series of β-NaYbF_4_:Er microrods were synthesized by a simple one-pot hydrothermal method and their intense green (545 nm) and red (650 nm) UCL were experimentally investigated based on the single-particle level under the excitation of 808 nm continuous-wave (CW) laser. Interestingly, the competition between the green and red UCL can be observed in highly Yb^3+^-doped microcrystals as the excitation intensity gradually increases, which leads to the UCL color changing from green to orange. However, the microcrystals doped with low Yb^3+^ concentration keep green color which is independent of the excitation power. Further investigations demonstrate that the cross-relaxation (CR) processes between Yb^3+^ and Er^3+^ ions result in the UCL competition.

**Graphical Abstract:**

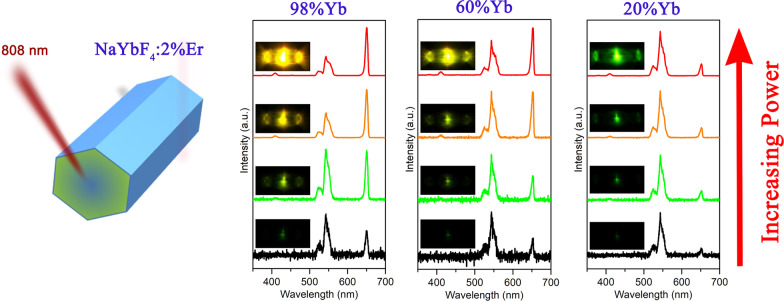

**Supplementary Information:**

The online version contains supplementary material available at 10.1186/s11671-021-03649-1.

## Introduction

Rare-earth-ion doped UC nanomaterials have drawn great attention recently due to their promising applications in biological issues [1], super-resolution imaging [2], multicolor display [3], thermometer sensor [4], laser refrigeration [5, 6], and laser materials [7, 8]. These UC nanomaterials can efficiently convert near-infrared light into visible emissions according to the anti-Stokes process. Generally, the achieving of UCL relies on the sensitizer–activator pair. To obtain the efficient UCL, the sensitizer–activator pair of lanthanides should incorporate in appropriate host lattices [9]. To date, the NaYF_4_ has been considered as the most efficient host for generating UCL owing to its low phonon energy (~ 350 cm^−1^) [10]. In general, the typical Yb^3+^ ions act as the sensitizer absorbing the excitation energy and the activator of Er^3+^ (Tm^3+^ or Ho^3+^) is responsible for emitting the UCL [11–13].

It is well-known that the Yb^3+^ ions have a large absorption cross-section at 980 nm, which can be efficiently excited by the high-performance and commercial laser diode [14]. However, owing to the large absorption coefficient of water molecules at 980 nm, the Yb^3+^-sensitized UC nanoparticles would face severe overheating problems, which limits its further application in biological tissues and aqueous environment by decreasing the depth of penetration [15]. To overcome the overheating effects, the conventional approach is to dope Nd^3+^ ions as sensitizer which can shift the excitation wavelength from 980 to 808 nm [16, 17]. Nonetheless, the dopant of Nd^3+^ usually yields small nanoparticles and hardly grows to microcrystals due to the larger Nd^3+^ (*r* = 1.249 Å) substitution of the relatively smaller Y^3+^ (*r* = 1.159 Å) in NaYF_4_ lattice [10]. Importantly, compared with the nano-scale UC particles, micro-scale UC particles facilitate more advantages for applications in micro-optoelectronic devices, volumetric color display, and microlasers based on their high crystallinity and luminescent efficiency [18–22]. However, the most present researches are mainly conducted in aqueous solutions, organic solvents or as-prepared solid powders. This may lead to severe overheating problems and the UCL will be influenced by the adjacent particles. Therefore, exploring the UCL and tunable color in single microparticle level, especially pumped at 808 nm wavelength, will effectively avoid the effects of external environment and broaden its further applications in micro-optoelectronic devices and aqueous environments.

In this study, we firstly report the effect of excitation-power-dependent UCL competition in single Yb^3+^-sensitized NaYbF_4_:Er microcrystal pumped at 808 nm. The properties of the UCL competition are characterized by the single microcrystal level. The competition between green and red UCL is clearly observed in highly Yb^3+^-doped microcrystals with varying the excitation intensity, and the UCL color was tuned from green to orange. On the contrary, there are no UCL competitions observed in lowly Yb^3+^-doped microcrystal and the UCL color always maintains green which is independent of the excitation power. The mechanism of the UCL competition is also demonstrated in detail.

## Experimental Sections

### Chemicals

The chemicals of yttrium nitrates (Y(NO_3_)_3_, 99.9%), ytterbium nitrates (Yb(NO_3_)_3_, 99.9%), erbium nitrates (Er(NO_3_)_3_, 99.9%), nitric acid (HNO_3_, analytical reagents), Ethylenediamine tetraaceticacid disodium salt dihydrate (EDTA-2Na, analytical reagents), sodium hydroxide (NaOH, analytical reagents) and ammonium fluoride (NH_4_F, analytical reagents) were purchased from Aladdin (China). All the chemicals were directly used as received without further purification.

### Synthesis of β-NaYF_4_:Yb,Er Microcrystals

The β-NaYF_4_:Yb,Er microcrystals were synthesized by a similar hydrothermal method procedure according to our previous study [23]. In a typical procedure, for instance, synthesis of the β*-*NaYbF_4_:2%Er (mol%) microcrystals: firstly, the Yb(NO_3_)_3_ and Er(NO_3_)_3_ powders were weighted according to the stoichiometric ratio and then dissolved in deionized water yielding a clear solution (0.2 mol L^−1^); then the EDTA-2Na (1 mmol) and NaOH (5 mmol) were mixed with 12.5 mL deionized water under continuously stirring in a beaker; following, 5 mL of Yb(NO_3_)_3_ (0.2 mol L^−1^) and Er(NO_3_)_3_ (0.2 mol L^−1^) aqueous solutions (the total Yb^3+^ and Er^3+^ ions are 1 mmol), 8 mL of NH_4_F (2 mol L^−1^) aqueous solutions and 7 mL of dilute hydrochloric acid (1 mol L^−1^) were added into the beaker; finally, the above mixtures were stirred for 1.5 h and transferred into a 50 mL Teflon-lined autoclave and heated at 200 °C for 40 h. The as-prepared white precipitates were collected by centrifugation, washed with deionized water and ethanol for several times, and dried in air at 40 °C for 8 h. The microcrystals doped with different concentrations of Yb^3+^ or Er^3+^ can be similarly synthesized by varying the volume of RE(NO_3_)_3_ aqueous solutions.

### Structural Characterization

The morphology and size of the β-NaYbF_4_:Er microcrystals were characterized by scanning electron microscope (SEM) (S4800, Hitachi). X-ray diffraction (XRD) patterns of the microcrystals were measured using a powder X-ray diffractometer with Cu K radiation at 40 kV and 200 mA (TTR III system, Rigaku).

### Upconversion Luminescence Measurements

In the photoluminescence experiments, the 808 nm CW laser (Mira-900F, Coherent) was integrated with an inverted microscope (Observer A1, Zeiss) and irradiated on the microcrystals with a 100× objective lens (NA = 1.4). The diameter of the excitation spot was estimated to be ~ 2.0 μm. The UCL generated from the microcrystals was collected by the same objective lens and then transmitted to a spectrometer (SR-500I-B1, Andor) coupled with a charge-coupled device (DU970N, Andor) for optical signal analysis. The UCL colors of the microcrystals were photographed using a high-sensitivity camera (DS-Ri2, Nikon).

## Results and Discussion

**Fig. 1:**
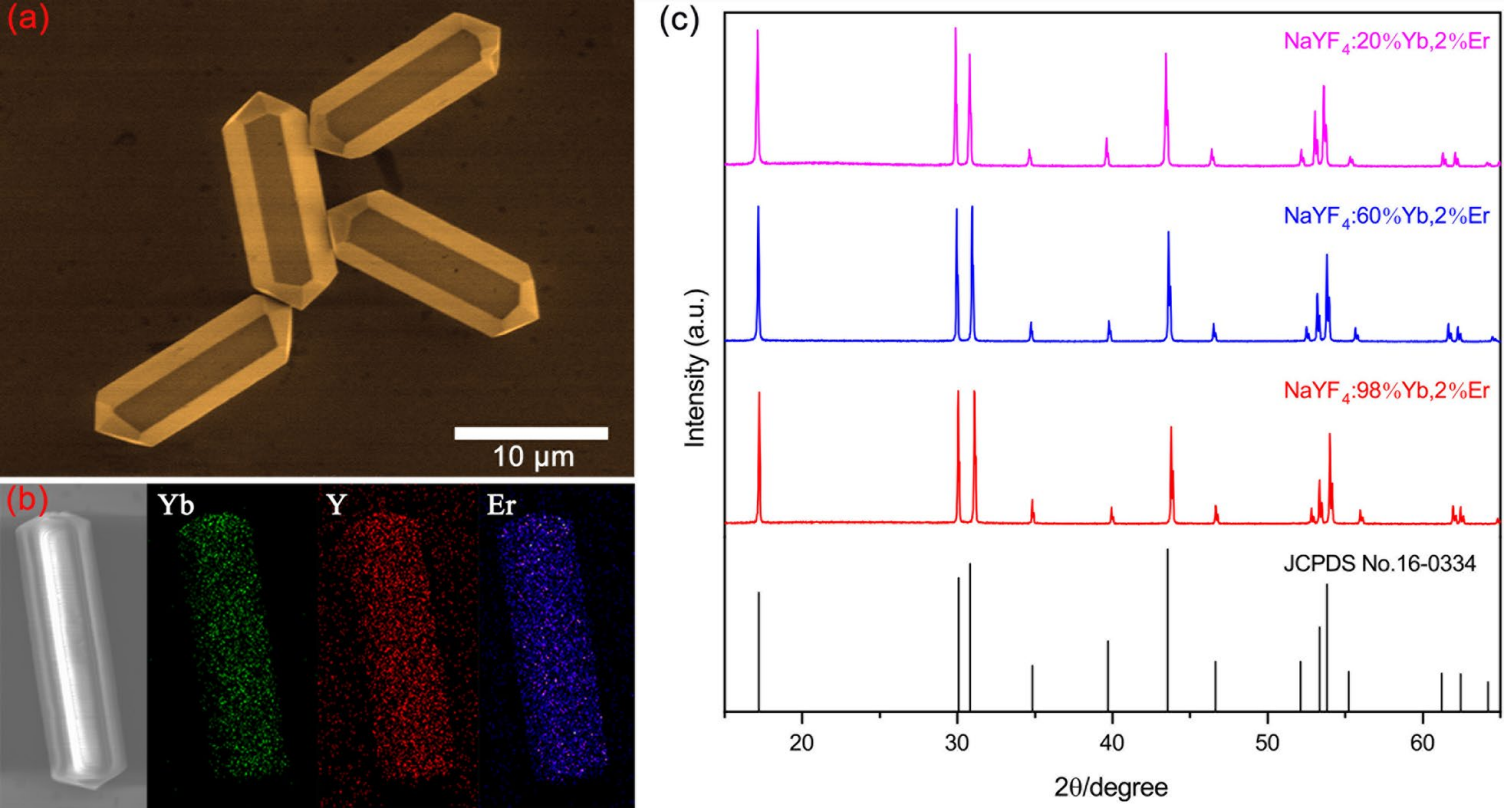
**a** SEM images of the β-NaYF_4_:98%Yb,2%Er microcrystals. **b** The elements mapping of β-NaYF_4_:60%Yb,2%Er microcrystals. **c** XRD patterns of the β-NaYF_4_:*x*%Yb,2%Er microcrystals

Figure 1a and Additional file 1: Fig. S1 show the SEM images of the as-prepared β-NaYbF_4_:Er microcrystals doped with different Yb^3+^ concentrations. The results indicate that the microcrystals exhibit the hexagonal prism morphology and uniform size distribution (with the lengths of ~ 15 μm and diameters of ~ 6 μm). Notably, adjusting the doping Yb^3+^ concentrations slightly varies the size of microcrystals. Figure 1b gives the elements mapping of single NaYF_4_:60%Yb,2%Er microcrystal, which demonstrates that the Y^3+^, Yb^3+^ and Er^3+^ ions are homogeneously incorporated in the NaYF_4_ host lattices. Figure 1c displays the XRD patterns of the β-NaYF_4_:*x*%Yb,2%Er microcrystals with different Yb^3+^ concentrations. It reveals that all the diffraction peaks are well in accordance with the standard hexagonal phases of NaYF_4_ host (JCPDS No. 16-0334). The SEM images and XRD patterns confirm that the NaYF_4_ microcrystals are successfully synthesized and highly crystalline.

**Fig. 2:**
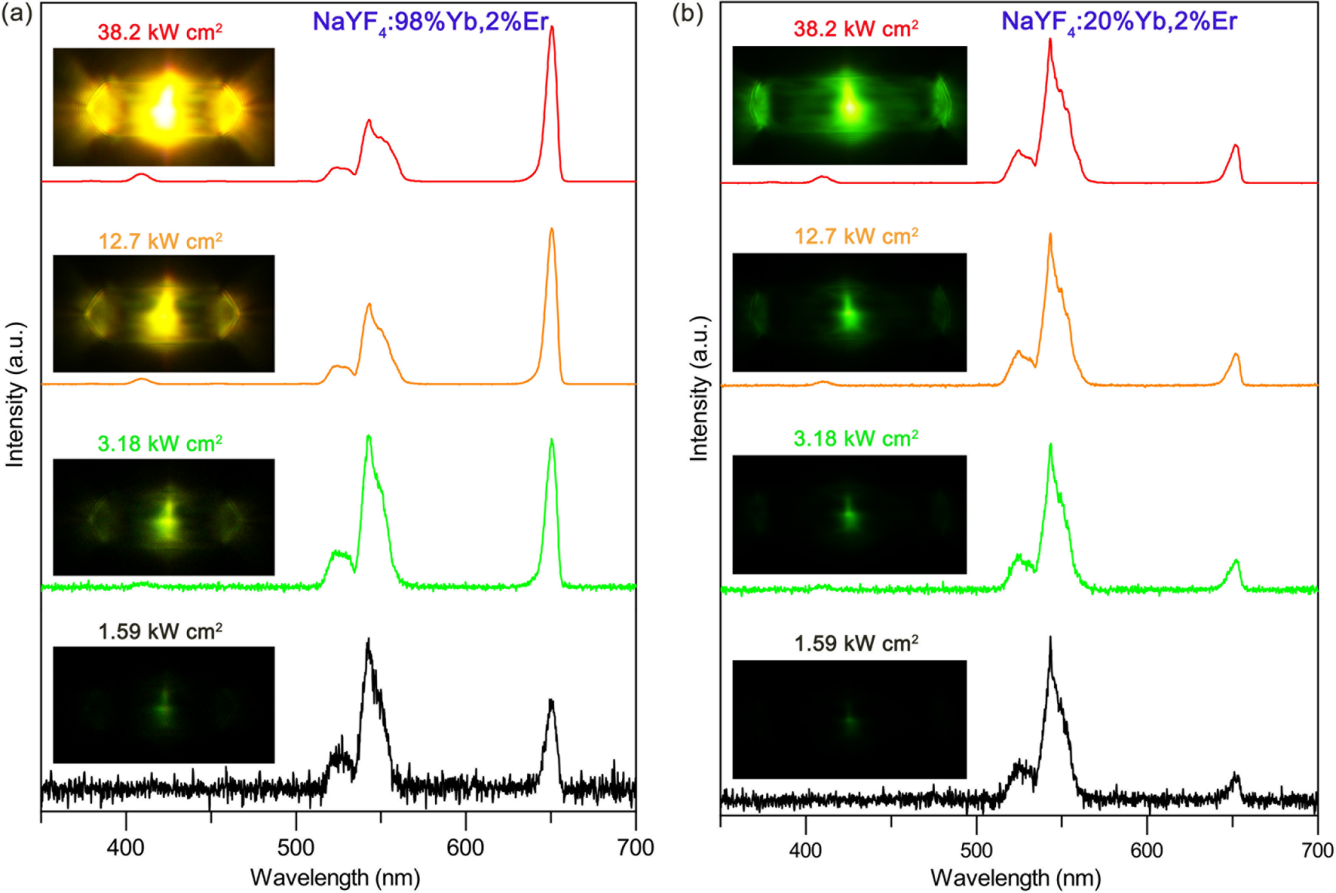
The UCL spectra of single **a** β-NaYF_4_:98%Yb,2%Er, and **b** β-NaYF_4_:20%Yb,2%Er microcrystal under the excitation of 808 nm CW laser with different excitation density. The insert UCL photographs are corresponding to the relevant spectrum, respectively

Figure 2 shows the UCL spectra of single β-NaYF_4_:*x*%Yb,2%Er microcrystal under the excitation of 808 nm CW laser with different excitation density. The corresponding UCL photographs are also provided in the inserts of relevant spectrum, respectively. Figure 2a gives the spectra of the highly Yb^3+^-doped single NaYbF_4_:2%Er microcrystal. The typical green (525 and 545 nm) and red (650 nm) UCL can be clearly observed, which are ascribed to the transitions of (^2^H_11/2_/^4^S_3/2_) → ^4^I_15/2_ and ^4^F_9/2_ → ^4^I_15/2_ from Er^3+^, respectively. Under the excitation density of 1.59 kW cm^−2^, the relatively weak green and red UCL emerge in the spectrum. The intensity of the green (545 nm) UC emission is larger than the red (650 nm) one, leading to the single hexagonal microcrystal exhibiting green color. As the excitation intensity slightly increases to 3.18 kW cm^−2^, the red UCL increases faster than the green UCL and their intensities are almost equal. This results in the UCL color changing to dark yellow. Notably, it can clearly observe that the UC emissions are transparent to the hexagonal microcrystal and transport from the middle of the microrod to the two side ports. This phenomenon has also been demonstrated in previous studies [21, 24]. When further rises the excitation intensity up to 12.7 kW cm^−2^, the red UCL enhances rapidly and exceeds the green UC emission, which causes the luminescence color tuning to yellow. Moreover, a new blue UCL centered at 410 nm appears, which is originated from the transition of ^2^H_9/2_ → ^4^I_15/2_ from Er^3+^. As the excitation intensity continues to increase to 38.2 kW cm^−2^, the red UCL increases remarkably and further surpasses the green UC emission leading to the UCL color turning into orange. The results demonstrate that the green and red UCL compete with each other as varies the excitation power. It is the first time that the UCL competition is observed in Er^3+^ ions for the highly Yb^3+^-doped micromaterials pumped at 808 nm.

To explore the influence of the Yb^3+^ concentration on the UCL competition, we further investigate the UCL properties of the single NaYF_4_:*x*%Yb,2%Er microcrystal doped with different Yb^3+^ concentrations. Additional file 1: Figure S2 displays the UCL spectra of the single NaYF_4_:60%Yb,2%Er microcrystal. The same phenomenon to the microcrystal doped with 98% Yb^3+^ can be observed (Fig. 2a). Differently, the red UCL exceeds the green UCL at a relatively high excitation intensity which is higher than that of the NaYF_4_:98%Yb,2%Er microcrystal. Moreover, the UCL color of the single NaYF_4_:60%Yb,2%Er microcrystal changes from green to yellow as the excitation intensity increases. However, as shown in Fig. 2b, when the doping Yb^3+^ ions further decrease to 20%, the green and red UCL keep a similar growth trend as the excitation intensity gradually reinforces. This leads to the UCL color maintaining green and no UCL competition occurs in the single microcrystal. Thus, the results verify that the highly Yb^3+^-doped microcrystals can efficiently generate the green and red UCL competition, causing the UCL color tunning from green to orange with increasing the excitation intensity. In contrast, for lowly Yb^3+^-doped microcrystals, the green UCL is always larger than the red one and the UCL color is green regardless of the variation of excitation intensity.

**Fig. 3:**
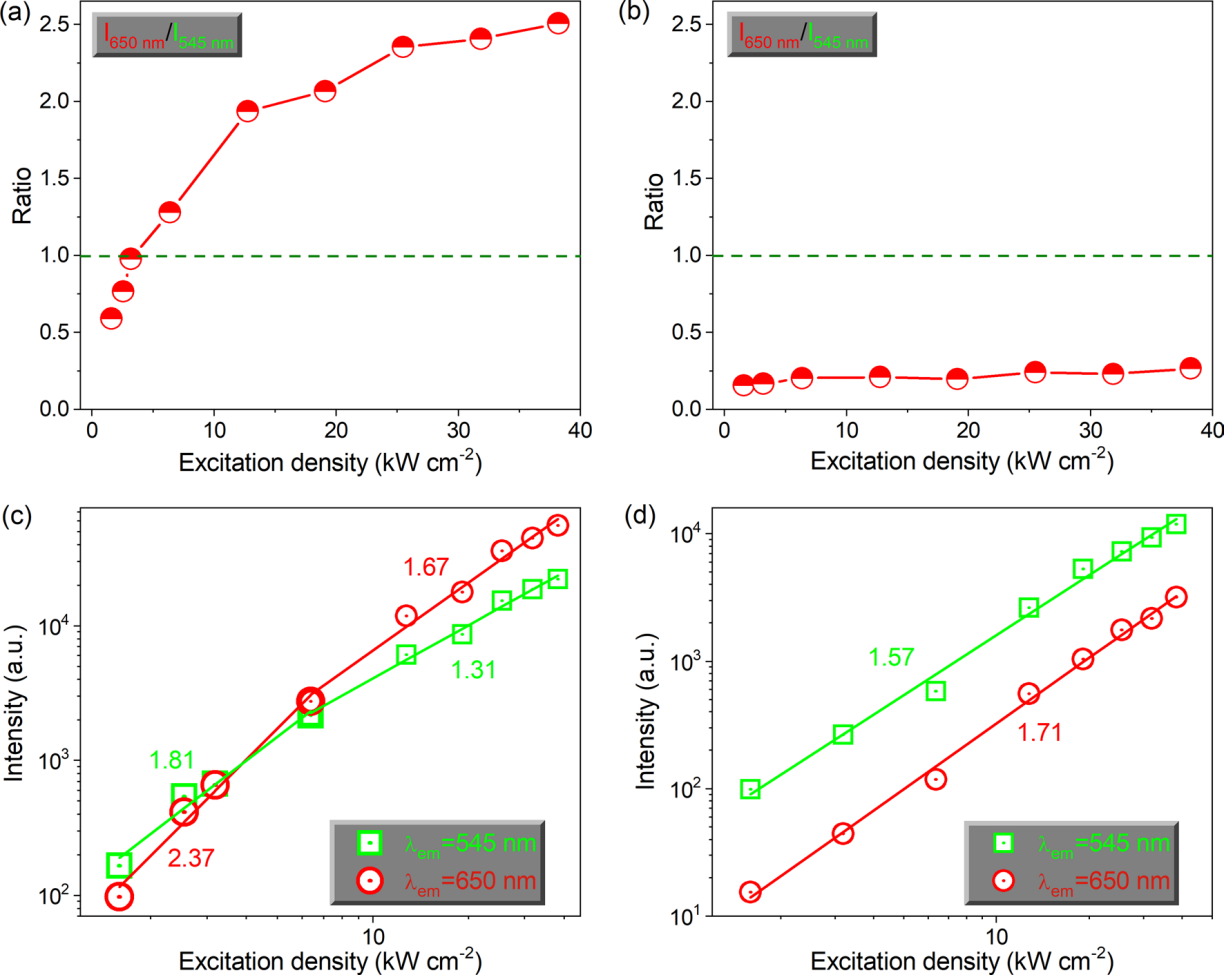
The ratios of *R*/*G* for single **a** β-NaYF_4_:98%Yb,2%Er, and **b** β-NaYF_4_:20%Yb,2%Er microcrystal as a function of the excitation intensity. The dependences of the UCL intensity on the excitation intensity for single **c** β-NaYF_4_:98%Yb,2%Er, and **d** β-NaYF_4_:20%Yb,2%Er microcrystal. All excitation wavelengths are at ~ 808 nm

To further investigate the UCL competition behaviors, we have calculated the ratios of red-to-green (*R*/*G*) UCL intensity based on the peak maxima for the single NaYF_4_:*x*%Yb,2%Er microcrystal under different pump powers, as shown in Fig. 3a, b. For NaYF_4_:98%Yb,2%Er microcrystal (Fig. 3a), the *R*/*G* ratios increase from 0.59 to 2.51 when the excitation intensity increases from 1.59 to 38.2 kW cm^−2^. Moreover, the red and green UCL are equivalent when the excitation intensity pumps at 3.18 kW cm^−2^. However, Additional file 1: Fig. S3a displays the *R*/*G* ratios rise merely from 0.19 to 1.36 as the excitation intensity gradually enhances. The excitation intensity for the red UCL exceeds the green one occurs at 12.7 kW cm^−2^. Exceptionally, for NaYF_4_:20%Yb,2%Er microcrystal shown in Fig. 3b, this *R*/*G* ratio keeps at ~ 0.20 which is independent of the excitation intensity. Figure 3c, d and Additional file 1: Fig. S3b show the dependences of UCL intensity on the excitation intensity for NaYF_4_:*x*%Yb,2%Er microcrystals doped with different Yb^3+^ concentrations. The slopes for the green and red UCL are all approximate to ~ 2, which indicates that these two UCL are derived from the two-photon absorption processes. Notably, for doping with 98% and 60% Yb^3+^ concentrations of microcrystals, the red and green UCL appear saturation effects. In addition, the excitation intensity for the NaYF_4_:98%Yb,2%Er microcrystal is lower than the NaYF_4_:60%Yb,2%Er microcrystal. However, the UCL slope of NaYF_4_:20%Yb,2%Er microcrystal is consistent under different excitation intensity due to without occurring saturation effects.

**Fig. 4:**
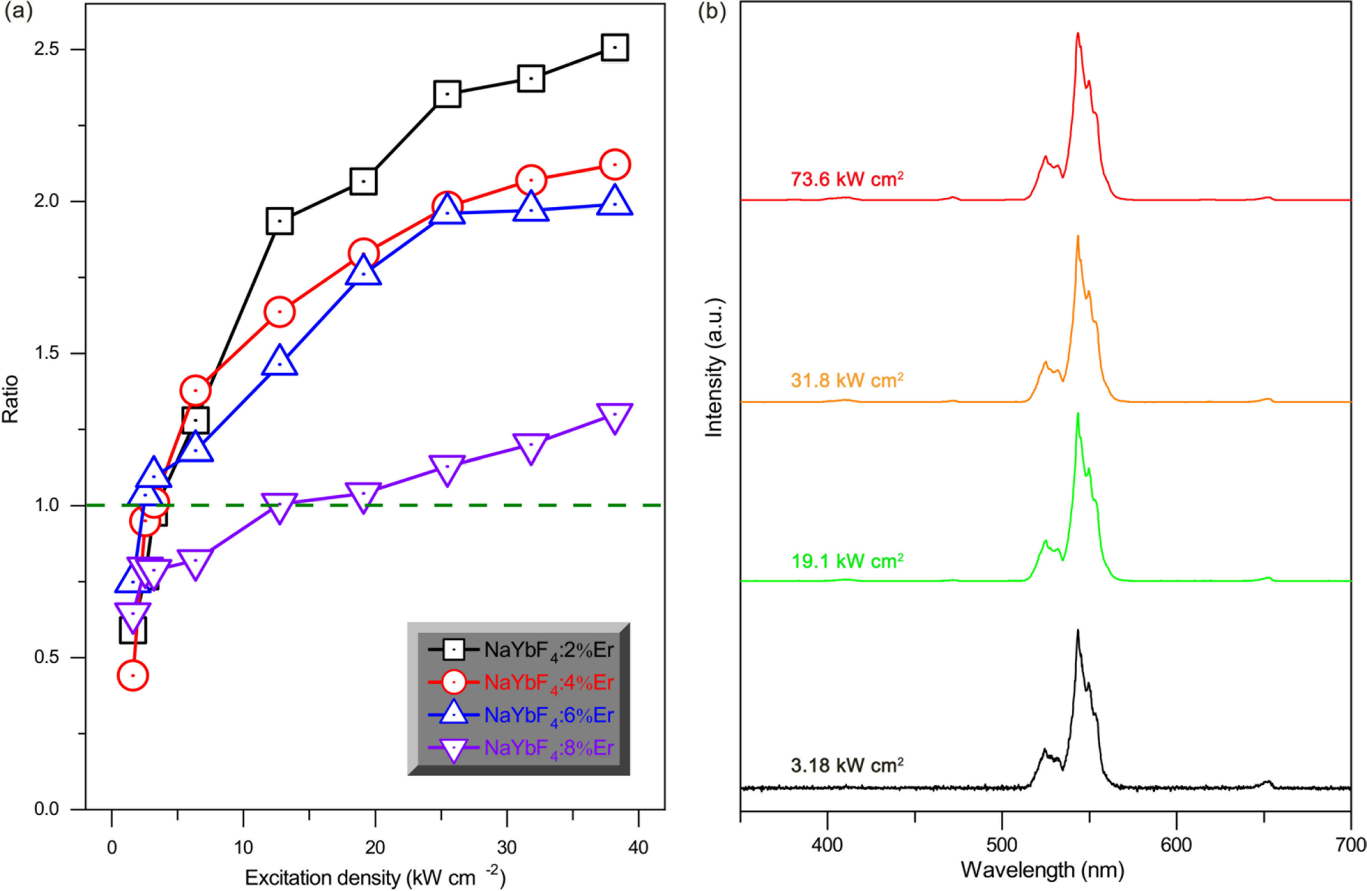
**a** The ratios of *R*/*G* for single β-NaYbF_4_:*x*%Er microcrystal doped with different Er^3+^ concentrations as a function of the excitation intensity. **b** The UCL spectra of single β-NaYF_4_:2%Er microcrystal under the excitation of 808 nm CW laser with different excitation density

Next, we further investigate the influence of doping Er^3+^ concentration on the UCL competition. Figure 4a illustrates the ratios of *R*/*G* as a function of the excitation intensity for single β-NaYbF_4_:*x*%Er microcrystals doped with different Er^3+^ concentrations. It reveals that the *R*/*G* ratios reduce as the doping Er^3+^ concentrations increase. Moreover, we make further efforts to explore the UCL properties for the single Er^3+^-doped NaYF_4_ microcrystal. Figure 4b shows the UCL spectra of single β-NaYF_4_:2%Er microcrystal under the excitation of 808 nm CW laser with different excitation density. The UCL spectra demonstrates that the green UCL is constantly larger than the red UCL and there is no UCL competition appearance. Therefore, its UCL color maintains green and is independent of the excitation intensity.

**Fig. 5:**
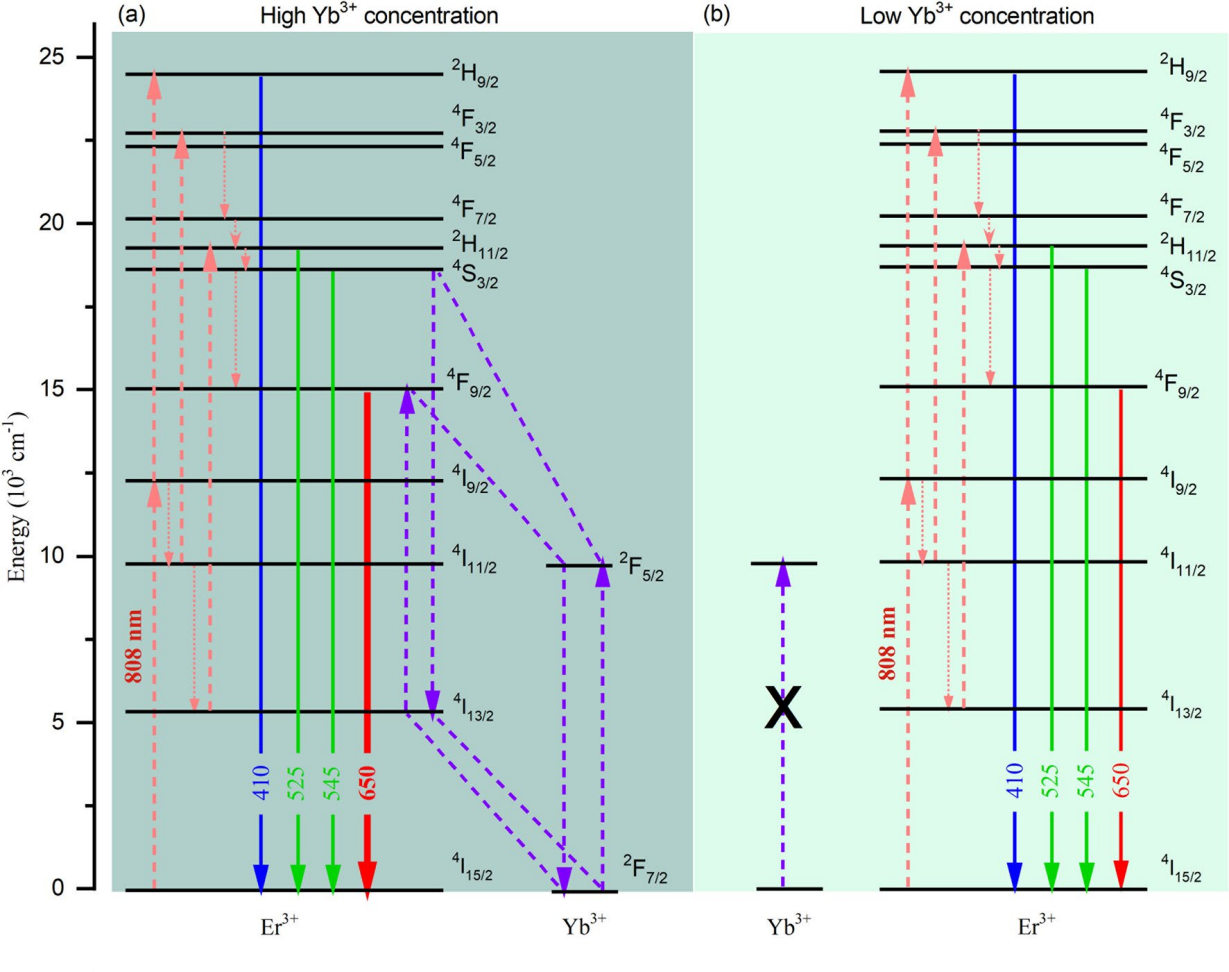
The proposed energy level diagram for **a** highly Yb^3+^-doped and **b** lowly Yb^3+^-doped β-NaYbF_4_:2%Er microcrystals, the corresponding mechanism of ET processes, radiative and non-radiative transitions are also provided

Having systematically demonstrated the experimental phenomenon, here we discuss the mechanism of the UCL competition induced by variation of excitation power. Figure 5 gives the proposed UCL mechanism and possible routes for populating the upper emitting states of Er^3+^ ions. The corresponding UCL transitions as well as the energy-transfer (ET) processes are also provided. The population of the Er^3+^ ions can be divided into two steps: firstly, the electrons in the ground state of Er^3+^ are excited to the ^4^I_9/2_ state by ground-state-absorption (GSA) or through ET from Yb^3+^ after absorbing the 808 nm photon; then continues to reach the ^2^H_9/2_ state by absorbing a second 808 nm photon or ^4^I_11/2_ state through a non-radiative transition [25]. After that, the emitting states (^2^H_11/2_, ^4^S_3/2_, and ^4^F_9/2_) can be populated by excited-state-absorption (ESA), CR, ET, and non-radiative transition processes, which are elaborated in Fig. 5. The significant population of the upper states of Er^3+^ ions can efficiently generate the UCL.

Notably, under the excitation of 808 nm laser, the doping Yb^3+^ concentrations will affect the populating routes for upper states of Er^3+^. For highly Yb^3+^-doped NaYF_4_:2%Er microcrystal, the distance between the Yb^3+^ and Er^3+^ is relatively close, thus leads to the significant CR processes happening. The proposed CR processes are:

1 $${\text{Er}}\left( {^{{4}} {\text{S}}_{{{3}/{2}}} ,^{{2}} {\text{H}}_{{{11}/{2}}} } \right) + {\text{Yb}}\left( {^{{2}} {\text{F}}_{{{7}/{2}}} } \right) \to {\text{Er}}\left( {^{{4}} {\text{I}}_{{{13}/{2}}} } \right) + {\text{Yb}}\left( {^{{2}} {\text{F}}_{{{5}/{2}}} } \right)$$

2 $${\text{Yb}}\left( {^{{2}} {\text{F}}_{{{5}/{2}}} } \right) + {\text{Er}}\left( {^{{4}} {\text{I}}_{{{13}/{2}}} } \right) \to {\text{Yb}}\left( {^{{2}} {\text{F}}_{{{7}/{2}}} } \right) + {\text{Er}}\left( {^{{4}} {\text{F}}_{{{9}/{2}}} } \right)$$

The above CR processes can efficiently enhance the population of red-emitting state (^4^F_9/2_) and depopulate the green-emitting states (^4^S_3/2_ and ^2^H_11/2_). Therefore, under relatively lower excitation intensity, it mainly populates the green-emitting states, thus the green UCL is larger than red one and the UCL color tends to be green. When gradually increasing the excitation intensity, the CR processes become efficient, thus enhancing the red UCL and suppressing green UC emissions. This causes the red and green UCL to compete with each other. This experimental phenomenon is similar to our previous literature reported that the highly-Yb^3+^ doped NaYF_4_:Er microcrystals always tend to generate red UCL color under 980 nm excitation [23]. However, for lowly Yb^3+^-doped NaYF_4_:2%Er microcrystal, there is no CR processes occurrence because of the relatively far distance between Yb^3+^ and Er^3+^ ions. Therefore, the green- and red-emitting states maintain the same populating proportion as increases the excitation intensity, which results in the microcrystal keeping green UCL color.

## Conclusions

In conclusion, we have systematically investigated the excitation power induced UCL competition in single NaYF_4_:*x*%Yb,2%Er microcrystal under the excitation of 808 nm. It finds that, for highly Yb^3**+**^-doped microcrystals, the red and green UCL compete with each other and its UCL color can be finely tuned from green to orange when gradually increasing the excitation intensity. On the contrary, there is no competition in lowly Yb^**3+**^-doped microcrystals and the UCL color retains green which is unchanged. The mechanism of the UCL competition is interpreted by CR processes owing to the short distance between Yb^3+^ and Er^3+^ ions in highly Yb^3+^-doped microcrystals. However, for lowly Yb^3+^-doped microcrystals, the long-distance between Yb^3+^ and Er^3+^ ions prohibits the CR processes and the population of Er^3+^ ions keeps the original approaches, thereby creating the lowly Yb^3+^-doped NaYF_4_:2%Er microcrystal facilitates green UCL color. Owing to the remarkable optical properties in micro-scale and particularly pumped at 808 nm laser, these microcrystals can be potentially applied in biological issues, aqueous environments and micro-optical devices.

## Supplementary Information

**Additional file 1: Fig. S1**. SEM images of the (a) β-NaYF4:60%Yb,2%Er, and (b) β-NaYF4:20%Yb,2%Er microcrystals. **Fig. S2**. The UCL spectra of single β-NaYF4:60%Yb,2%Er microcrystal under the excitation of 808 nm CW laser with different excitation density. The insert UCL photographs are corresponding to the relevant spectrum, respectively. **Fig. S3**. (a) The ratios of R/G for single β-NaYF4:60%Yb,2%Er microcrystal as a function of the excitation intensity. (b) The dependences of the UCL intensity on the excitation intensity for single β-NaYF4:60%Yb,2%Er microcrystal. All excitation wavelengths are at ~808 nm. **Fig. S4**. The microscope image of well dispersed β-NaYF4:60%Yb,2%Er microcrystals. Fig. S5 The microscope image of a single β-NaYF4:60%Yb,2%Er microcrystal excited by the 808 nm CW laser. **Fig. S6**. The microscope image of single β-NaYF4:60%Yb,2%Er microcrystals excited by the 980 nm CW laser without illustrated light.

## Data Availability

The datasets used and/or analyzed during the current study are available from the corresponding author on reasonable request.

## Abbreviations

UCL: Upconversion luminescence
CW: Continuous-wave
CR: Cross-relaxation
SEM: Scanning electron microscopy
XRD: X-ray diffraction
*R*/*G*: Ratio of red-to-green
ET: Energy transfer
GSA: Ground-state-absorption
ESA: Excited-state-absorption
