# Supplementary material for: Excitation-Power-Dependent Upconversion Luminescence Competition in Single β-NaYbF4:Er Microcrystal Pumped at 808 nm
Source: Nanoscale Res Lett. 2022 Mar 26;17:38. doi: 10.1186/s11671-021-03649-1 (PMC8964848; doi:10.1186/s11671-021-03649-1)
Supplement: Supplementary file 1 — Additional file 1: Fig. S1. SEM images of the (a) β-NaYF4:60%Yb,2%Er, and (b) β-NaYF4:20%Yb,2%Er microcrystals. Fig. S2. The UCL spectra of single β-NaYF4:60%Yb,2%Er microcrystal under the excitation of 808 nm CW laser with different excitation density. The insert UCL photographs are corresponding to the relevant spectrum, respectively. Fig. S3. (a) The ratios of R/G for single β-NaYF4:60%Yb,2%Er microcrystal as a function of the excitation intensity. (b) The dependences of the UCL intensity on the excitation intensity for single β-NaYF4:60%Yb,2%Er microcrystal. All excitation wavelengths are at ~808 nm. Fig. S4. The microscope image of well dispersed β-NaYF4:60%Yb,2%Er microcrystals. Fig. S5 The microscope image of a single β-NaYF4:60%Yb,2%Er microcrystal excited by the 808 nm CW laser. Fig. S6. The microscope image of single β-NaYF4:60%Yb,2%Er microcrystals excited by the 980 nm CW laser without illustrated light. [file 11671_2021_3649_MOESM1_ESM.doc]

**Supplementary material**

Excitation-power-dependent upconversion luminescence competition in single β-NaYbF_4_:Er microcrystal pumped at 808 nm

Maohui Yuan,^1,4,6^ Zining Yang,^1,2,3,6^ Xu Yang,^1,2,3^ Linxuan Wang,^1,2,3^ Rui Wang,^1,2,3^ Sheng Lan,^5^ Kai Han,^1,2,3,*^ Hongyan Wang,^1,2,3,*^ and Xiaojun Xu^1,2,3^

*^1^College of Advanced Interdisciplinary Studies, National University of Defense Technology, Changsha, 410073, China*

*^2^State Key Laboratory of Pulsed Power Laser Technology,* *National University of Defense Technology, Changsha, 410073, China*

*^3^Hunan Provincial Key Laboratory of High Energy Laser Technology, National University of Defense Technology, Changsha, 410073, China*

*^4^Department of physics and chemistry, PLA Army Academy of Special Operations, Guangzhou 510507, China*

*^5^Guangdong Provincial Key Laboratory of Nanophotonic Functional Materials and Devices, School of Information and Optoelectronic Science and Engineering, South China Normal University, Guangzhou, 510006, China*

*^6^Maohui Yuan and Zining Yang contributed equally to this work.*

**Corresponding author:* [*hankai0071@nudt.edu.cn*](mailto:hankai0071@nudt.edu.cn) *and wanghongyan@nudt.edu.cn*


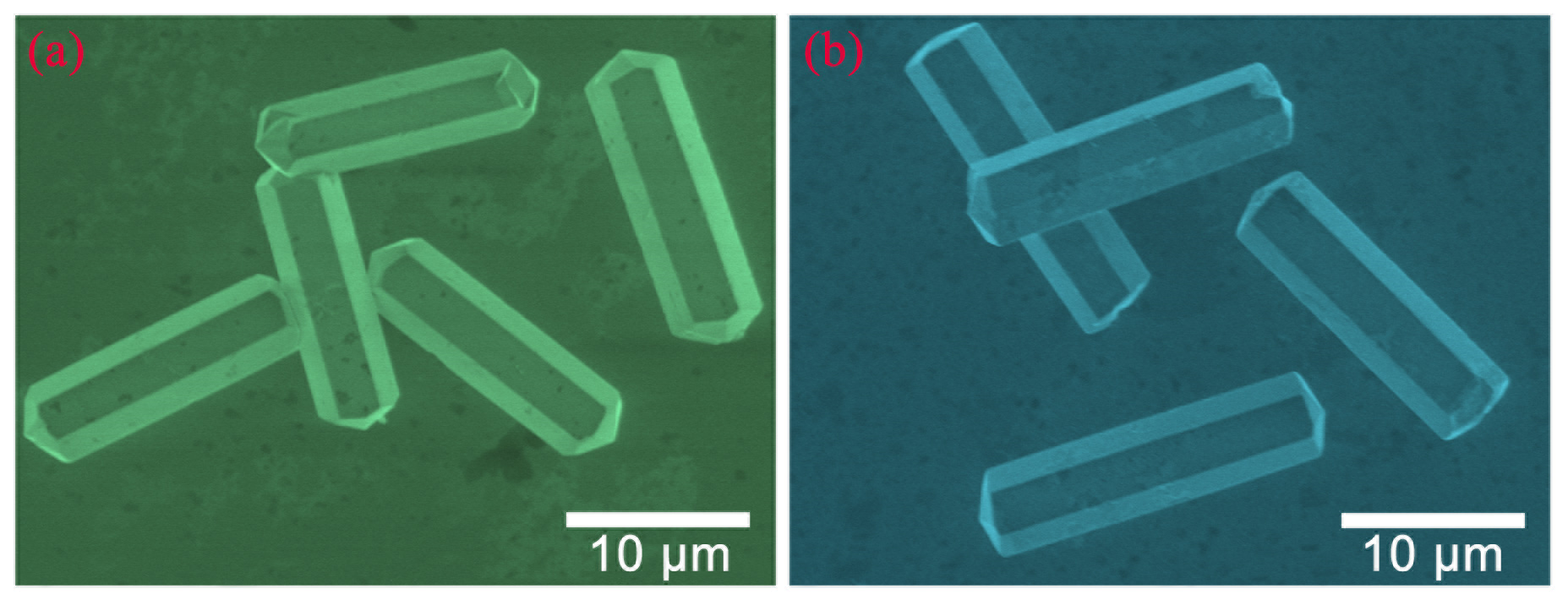


Fig. S1. SEM images of the (a) β-NaYF_4_:60%Yb,2%Er, and (b) β-NaYF_4_:20%Yb,2%Er microcrystals.


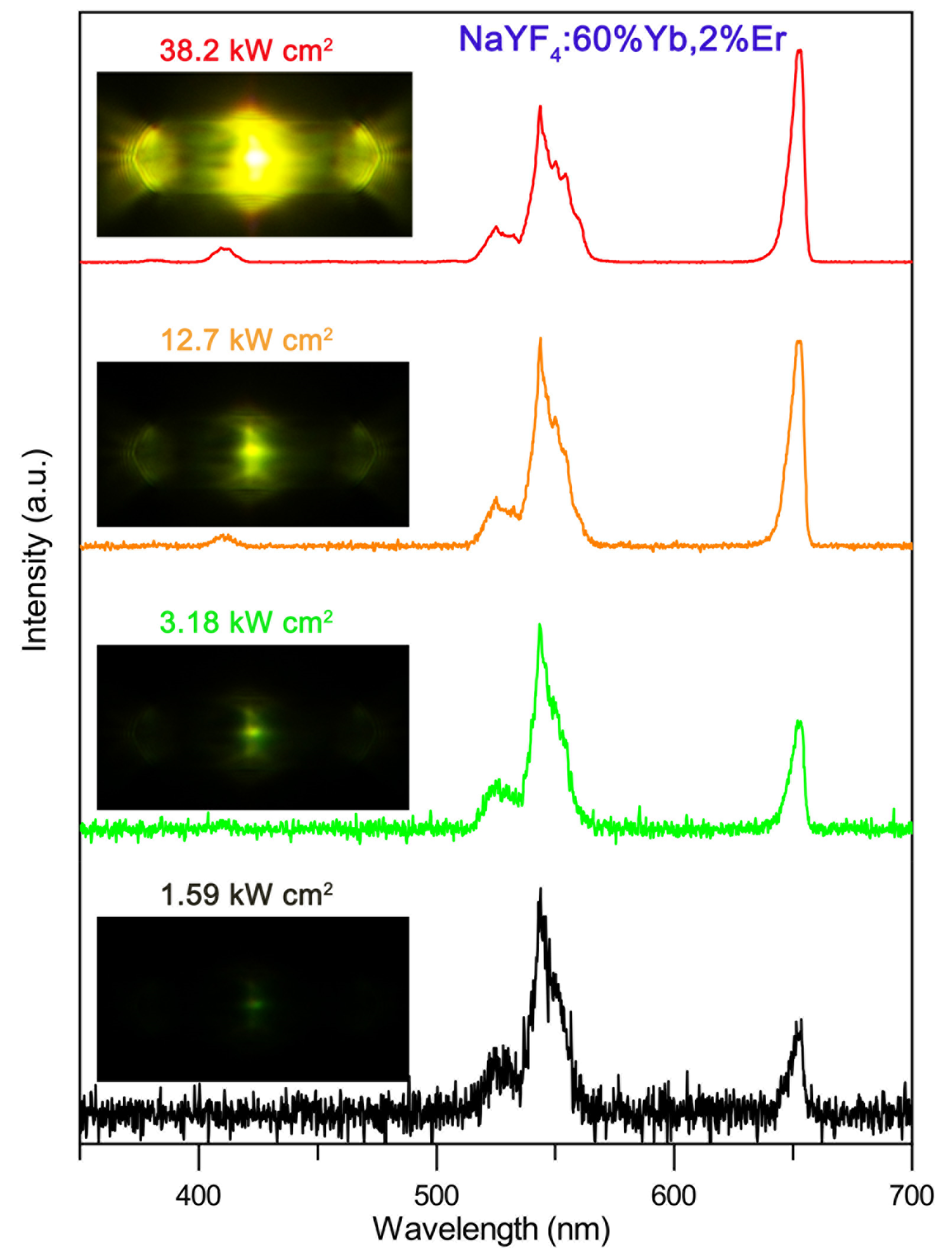


Fig. S2. The UCL spectra of single β-NaYF_4_:60%Yb,2%Er microcrystal under the excitation of 808 nm CW laser with different excitation density. The insert UCL photographs are corresponding to the relevant spectrum, respectively.


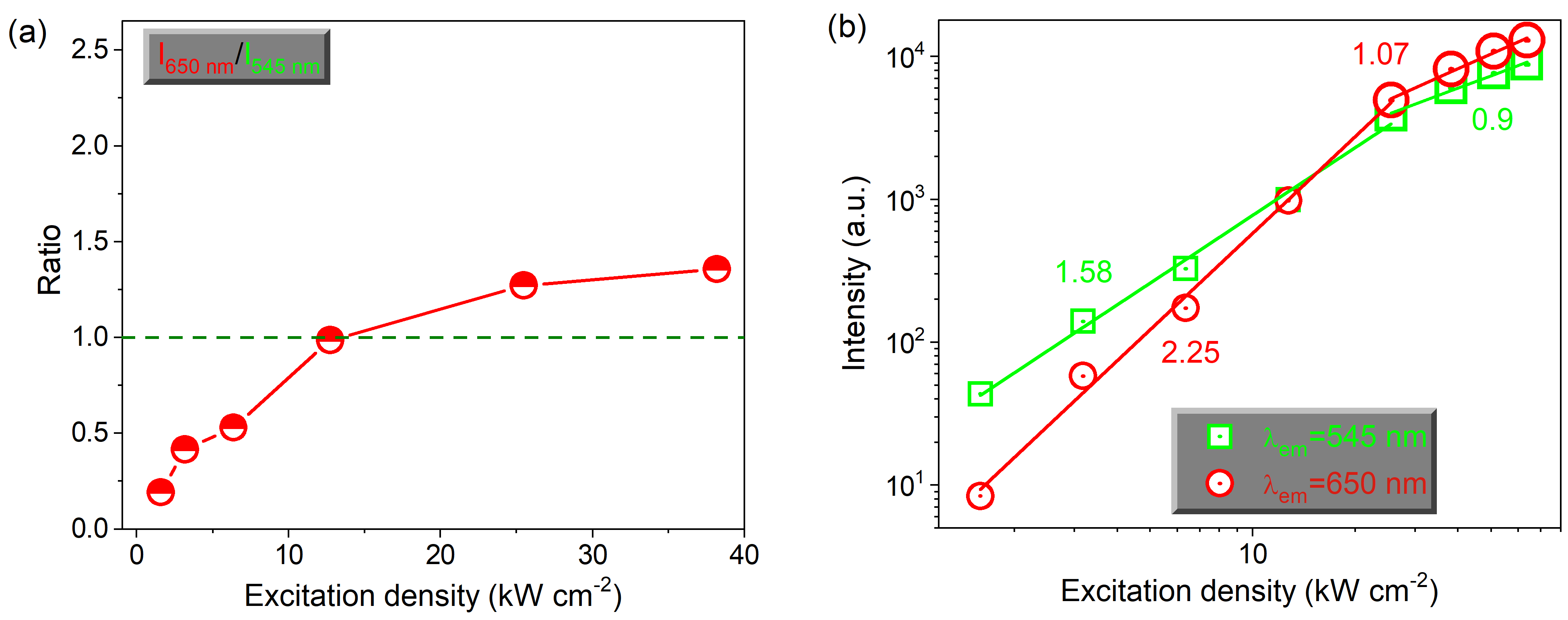


Fig. S3. (a) The ratios of R/G for single β-NaYF_4_:60%Yb,2%Er microcrystal as a function of the excitation intensity. (b) The dependences of the UCL intensity on the excitation intensity for single β-NaYF_4_:60%Yb,2%Er microcrystal. All excitation wavelengths are at ~808 nm.


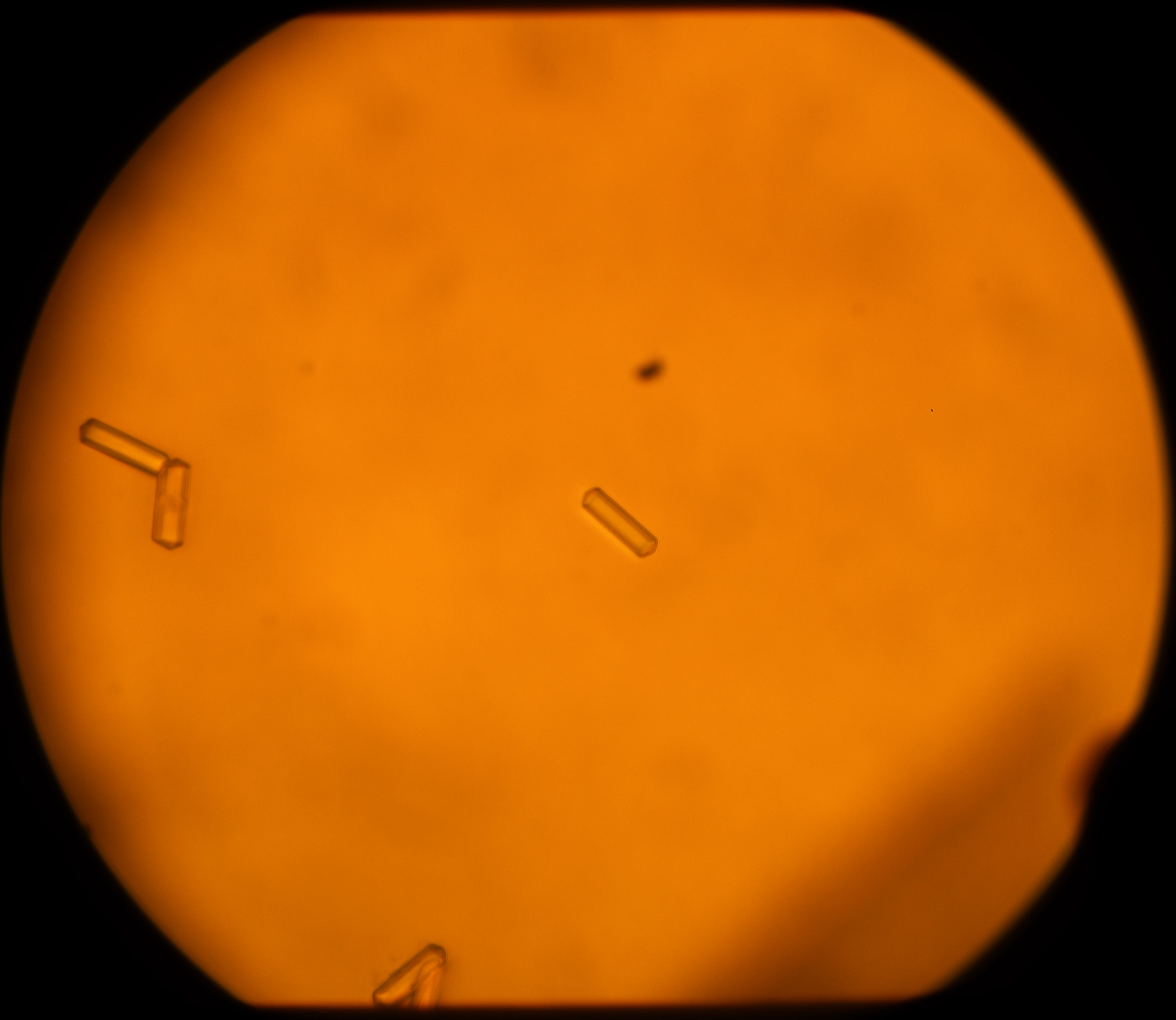


Fig. S4. The microscope image of well dispersed β-NaYF_4_:60%Yb,2%Er microcrystals.


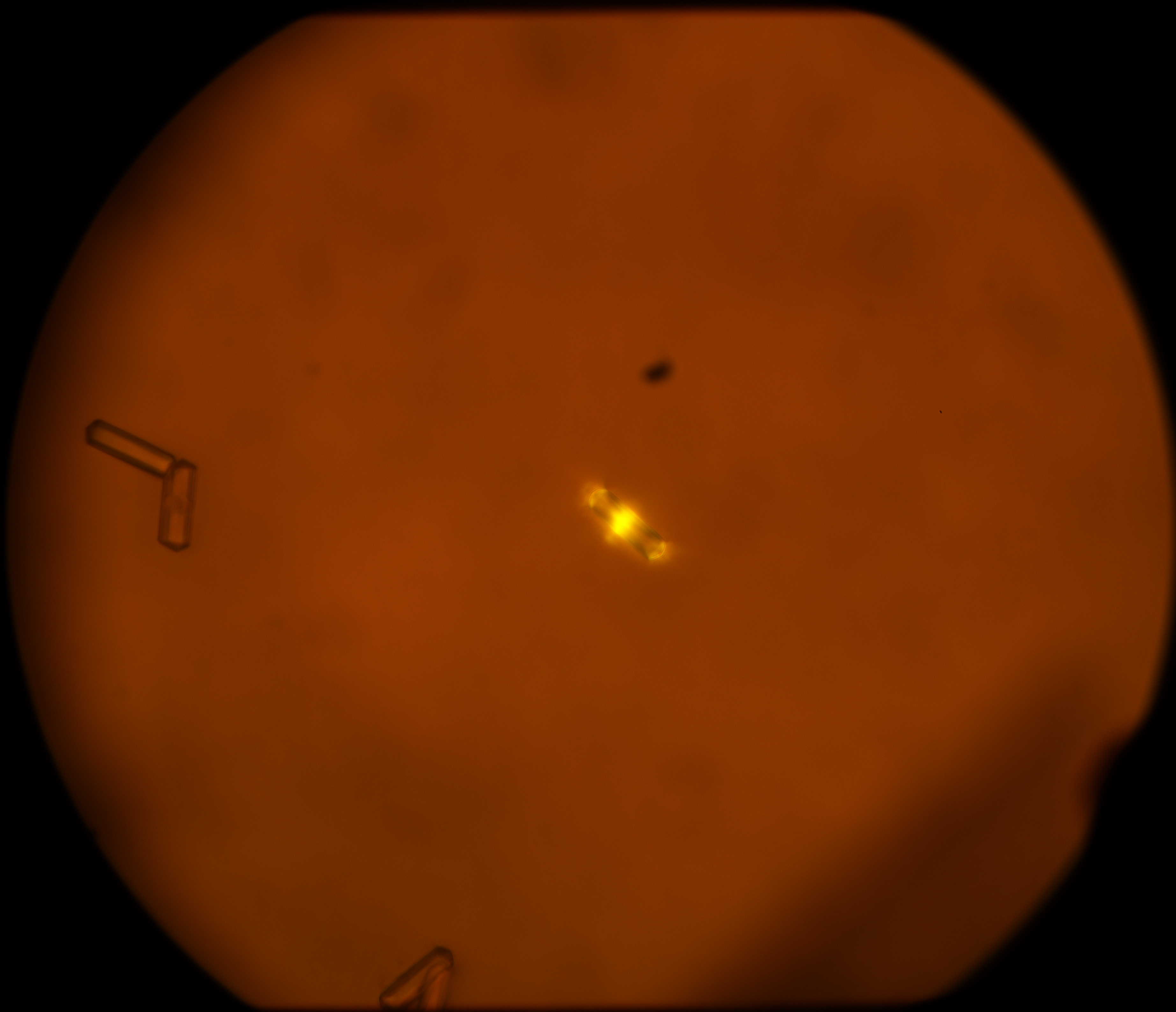


Fig. S5 The microscope image of a single β-NaYF_4_:60%Yb,2%Er microcrystal excited by the 808 nm CW laser.


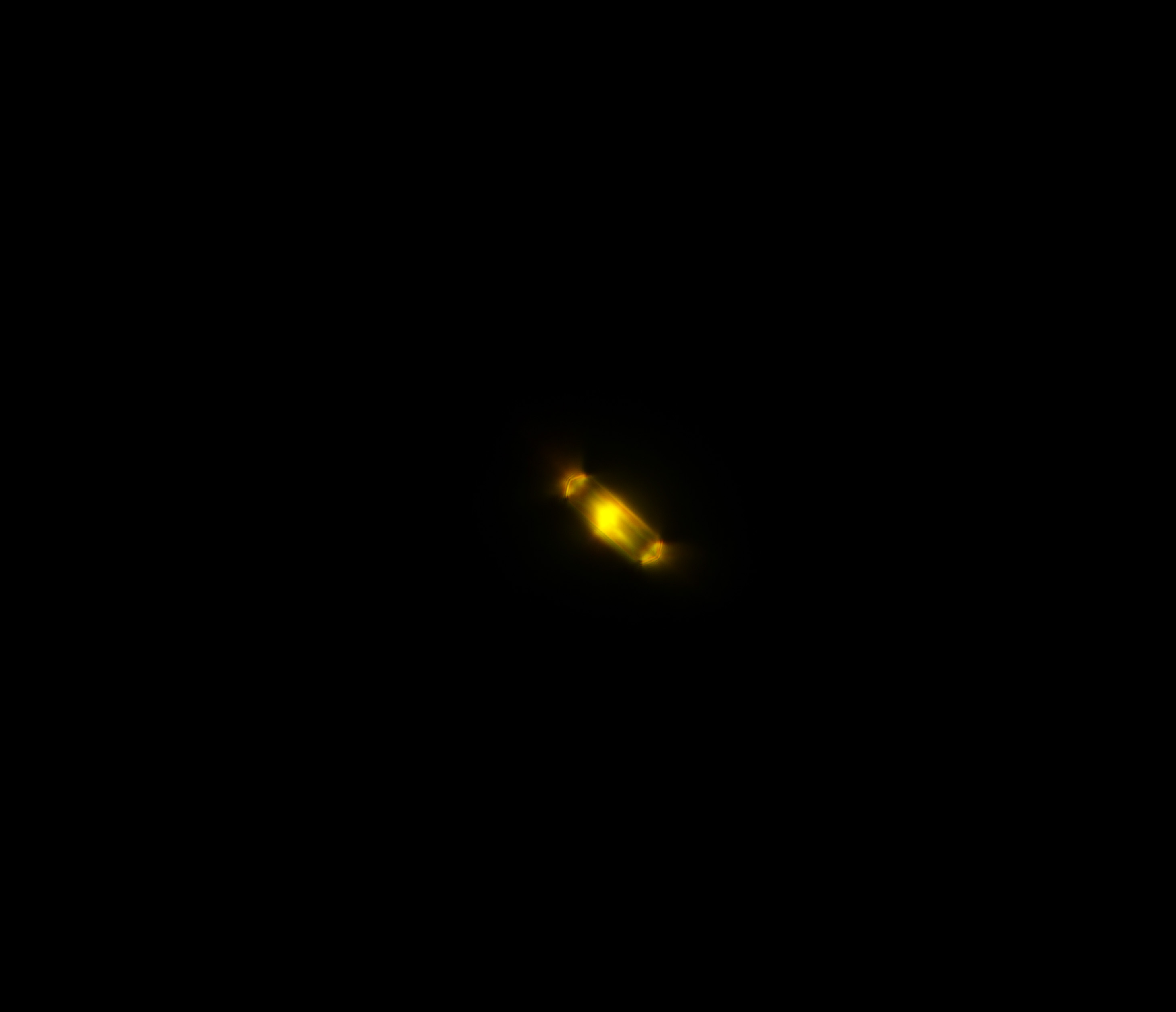


Fig. S6. The microscope image of single β-NaYF_4_:60%Yb,2%Er microcrystals excited by the 980 nm CW laser with illustrated light.
